# Knowledge, Attitudes, and Awareness of New Jersey Public High School Students about Concepts of Climate Change, including Environmental Justice

**DOI:** 10.3390/ijerph20031922

**Published:** 2023-01-20

**Authors:** Derek G. Shendell, Lily F. Black, Yvette Way, Juhi Aggarwal, Maryanne L. F. Campbell, Kimberly T. Nguyen

**Affiliations:** 1 New Jersey Safe Schools Program (NJSS), Rutgers School of Public Health (SPH), 683 Hoes Lane West, 3rd Floor SPH-Suite 399, Piscataway, NJ 08854, USA; 2 Department of Environmental and Occupational Health and Justice, Rutgers School of Public Health (SPH), Piscataway, NJ 08854, USA

**Keywords:** adolescents, climate change, environmental justice, science education, sustainability

## Abstract

Increasing acknowledgement of climate change (CC) has encouraged various responses, such as education standard mandates. In 2021, New Jersey (NJ) became the first U.S. state to require K–12 CC education across subjects, effective fall 2022. This necessitated introductory science courses on CC to support high school (HS) curricula. Thus, NJ Safe Schools Program (NJSS) created a new course titled, “Introduction to HS Students to CC, Sustainability, and Environmental Justice (EJ).” Given that the COVID-19 pandemic continues (2020–2023 school years) and vaccination coverage varies, this course was developed and approved in an asynchronous online format. Its five modules cover environmental science, CC, natural disasters and extreme weather events, sustainability, including energy conservation and efficiency definitions, and EJ. A 20-question survey included at the end, modified/adapted from a larger nationwide U.S. Student Conservation Association (SCA) survey 2019–2020, examined the perspectives of HS students concerning CC. Selected volunteer NJ HS enlisted students (n = 82/128 finished) to pilot this course February–April 2022. Results such as average scores ≥90% suggested success regarding initial knowledge and awareness gained; for individual modules, two knowledge checks >80% and three knowledge checks >90%. The SCA survey results, overall and by region in NJ, highlighted how most students felt about CC and extreme weather events, plus issues such as EJ. This NJSS introductory course opened in July 2022 for NJ public county secondary school districts and comprehensive HS with approved career-technical education programs, and potentially elsewhere.

## 1. Introduction

In 2020, the State of New Jersey (NJ) Department of Education and First Lady Tammy Murphy announced NJ would require climate change education across subject areas in grades K–12 to increase knowledge about the environment, how it interacts with life, and the changes resulting from climate change [1,2,3]. Originally slated to begin in 2021, there was a one-year delay to fall 2022 (2022–2023 school year) due to the COVID-19 pandemic [1,2,3]. In doing so, NJ became the first state in the United States (U.S.) to require climate change education across grade levels and subject areas [1,2,3]. Due to this new climate change awareness and education initiative from the State of NJ, there was a clear need for an increase in teaching students, including high school (HS) students, the basics of environmental science and environmental health to stimulate further education and thought. It was also important to know the current state of knowledge, awareness, and opinions or attitudes of HS students with respect to climate change and environmental justice (EJ) based on various sources of information within and outside of formal and informal education prior to the formal, official implementation of the updated State of NJ standards. A recent scoping review focused on information sources for teachers, not students, and how they used the internet, government agencies, media, and professional development. [4].

Worldwide, citizens, communities, and governments accept the role climate change plays in altering ecosystems and environmental quality. Major world leaders and thousands of participants joined together for the annual United Nations (U.N.) Climate Change Conferences. The most recent two held during the time period relevant to our project in NJ were in Glasgow (COP26) in October–November 2021 and in Egypt (COP27) in November 2022. Thus, the State of NJ has a forward-thinking education and the idea that the world needs younger generations to be empowered, willing, and able to combat climate change.

Environmental science education and the acceptance of scientifically sound climate change research are fundamental pillars of a sustainable future [1]. Due to politics, climate change and science in general have been confronted; the need for tested, science-based educational curricula to discredit false narratives of anti-climate change media is greater than ever in U.S. public school systems [4,5,6]. In the last decade, there has been a growing body of peer-reviewed literature related to climate change and its associations with human and ecological health and safety-related issues, including EJ. However, to date, excluding studies and reviews about educating adults in colleges/universities and groups of professionals such as health care providers, there has been limited peer-reviewed research in English specifically about educating HS students [7,8,9,10,11,12,13,14,15,16,17,18,19,20,21,22,23].

A unique aspect of environmental science education is how it is multi-faceted, diverse, broad, and applicable in a wide range of other disciplines. For example, the impacts of the economy, public health, nature of politics, etc., either depend on the environment, can impact the environment, or both. Environmental science education can inspire individuals and help inform a future where the environment is always taken into consideration, i.e., “behaving pro-environmentally” [24]. To behave pro-environmentally is to not put the climate and environmental quality of planet Earth completely at the mercy of anthropogenic forces. Ardoin et al. [25] reported that environmental science education has the greatest learning outcomes when coupled with other subjects and disciplines such as biology, chemistry, math, social studies, etc.

Work-based learning (WBL) and career and technical education (CTE), sometimes called career-technical-vocational education, are terms coined by the Carl D. Perkins Career and Technical Education Act of 2006, which was amended by the Strengthening Career and Technical Education for the 21st Century Act (Perkins V), to explain how specific education can be used to prepare students for life after school [26]. The knowledge provided in courses can assist participating HS students in future learning, internships, and varying work environments/conditions. Moreover, Ardoin et al. [25] also concluded environmental science education is positively related to academic achievement and civic engagement, i.e., it can help future generations excel in any career cluster and program pathway selected by a student.

An equally relevant aspect of environmental science education in modern society is teaching younger generations about environmental (in)justice. Empowering children through social, political, and academic routes can help create a more well-rounded approach to EJ education and promote critical thinking and problem solving [27]. Only about 20% of the public is scientifically literate [28]. Expanding the education of children and increasing scientific literacy not only involves the protection of the environment but also promotes equity in and access to education and environmental health. Indeed, the seventeen pillars of EJ [29] created in 1991 are still upheld today as necessary measures needed to reach EJ in the U.S. Thus, younger generations need to be aware and mindful of EJ and the known and potential injustices observed throughout our communities. With hundreds of years of unawareness and neglect in our lower-income and/or ethnic minority communities being recognized, leaders and policymakers need to prevent further harm. By educating school-age children about EJ, they could one day become leaders to create better futures for those experiencing the greatest consequences of pollution and its cumulative impacts. Regardless of career path or education status, an improved understanding of EJ and efforts related to addressing the impacts of EJ are needed to promote health, safety, and environmental quality at local, state, regional, and national levels.

In this paper, we describe how the NJ Safe Schools Program (NJSS) created a new five-module course titled “Introduction to HS Students to Climate Change, Sustainability, and EJ” and the results of the initial beta-to-pilot testing of this course in the 2021–2022 school year. Given the COVID-19 pandemic continues (2020–2023 school years) and vaccination coverage varies, this course was developed and approved in an asynchronous online format. The course covers concepts in environmental science, climate change, sustainability, including energy conservation and efficiency definitions, natural disasters and extreme weather events, and EJ. For national disasters and extreme weather events, we included concepts and definitions related to occupational safety and health, given the risks during cooling season months due to heat stress and during heating season months due to cold stress and the related illnesses and injuries [30,31,32,33]. Each of the five modules ends with a knowledge check activity, which is formatted like a quiz. At the end of the course, students completed a course reflection activity, an overall course evaluation, and a 20-question survey. This survey was modified/adapted from a larger nationwide U.S. Student Conservation Association (SCA) survey 2019–2020 on the perspectives of HS students concerning climate change [34]. Thus, this paper includes data on the initial knowledge, attitudes or beliefs, and awareness of these participating NJ HS students regarding climate change concepts, including EJ and sustainability, in the school year prior to the formal implementation of new NJ mandates [1,2,3].

## 2. Materials and Methods

This environmental science education course, created through the New Jersey Safe Schools Program (NJSS) within the Rutgers School of Public Health (SPH), occurred during the fall and early winter of 2021 to eventually be approved by the NJ Department of Education (NJDOE) in spring 2022 for future school years starting in 2022–2023. The course is offered asynchronously to HS students through the Rutgers Canvas Learning Management System (Canvas LMS). The course will serve as a supplemental education opportunity to support the NJDOE’s implementation of NJ Student Learning Standards or goals concerning climate change (including natural disasters and extreme weather events), sustainability, and environmental justice/cumulative impacts. The course was constructed with modules containing background information on environmental science, climate change, sustainability, extreme weather events and natural disasters, and EJ (and social justice) efforts. We determined these topics would be beneficial for students in their future careers across clusters and program pathways.

### 2.1. Study Population Samples

The course’s beta, internal, and pilot testing occurred in the winter of 2021 and the early spring of 2022. A combined “Rutgers-beta” testing analysis was performed with NJSS employees, Rutgers undergraduate students with varying majors, and MPH candidates with a variety of concentrations in Winter 2021. Minor adjustments were made with their feedback, but no substantial changes were needed, which allowed the course to proceed to the next test phase. Pilot testing began in February 2022 with three HS: Cinnaminson HS (CHS, S. NJ-1) Advanced Placement environmental and biology students (parts of STEM or science, technology, engineering, and mathematics education); co-op and non-STEM West Deptford HS (WDHS, S. NJ-2) students; and cosmetology students from Somerset County Vocational Technical HS (SCVTHS, C. NJ-1). These three schools completed the course between February and March 2022; data were collected until early April 2022. After pilot testing, per NJDOE approval, the course was available to HS throughout NJ by summer and fall 2022 for the 2022–2023 school year.

### 2.2. Course Design and Content including Modules Assessments

**Figure 1:**
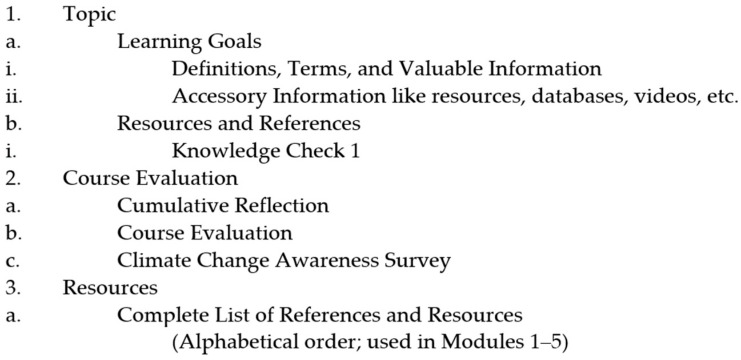
Example outline of a module scene in the course.

This concise introductory science course was envisioned to take NJ HS students about two hours or three NJ HS class periods to complete through an online learning platform, the Canvas LMS at Rutgers, The State University of NJ. The course consisted of a modular outline: (1) Background on Environmental Science, (2) Climate Change, (3) Natural Disasters/Extreme Weather Events, (4) Sustainability, (5) Environmental Justice Concepts, and ended with the Course Evaluation. Each module was organized as follows: learning goals (for each topic), definitions, terms, and valuable information, accessory pertinent information, resources and references, and a knowledge check (Figure 1). Each knowledge check had a total of five questions—four multiple choice and one open-ended—giving students an opportunity to brainstorm and apply the material they had just learned. The open-ended questions were graded individually based on completion. Knowledge checks were graded to show accuracy but did not count towards the final grade as they were only intended to serve as a marker for how much the students are learning and retaining (initial impact data).

Please see 2021–2022 module knowledge checks, with questions and answers, in Supplementary Materials as Figures S1–S5.

It was important for the accuracy and reliability of the course to use reputable global, federal, and state government resources such as President Biden’s White House Briefing on the climate crisis [35], the U.S. Environmental Protection Agency’s (EPA) information on Climate Change Indicators [36], the United Nations (UN) description of COP meetings [37], the NJDOE climate change initiative webpage [1], etc. Another goal of using such information sources was to provide the students with awareness of valuable tools to continue their own education. Other sources such as footprintcalculator.org [38] were provided so students could take the knowledge they have gained from the course and initially apply it to their lives outside of school and work in a fun, engaging, interactive activity.

### 2.3. Course Evaluation and SCA Survey

The course evaluation section initially consisted of three surveys. Two surveys were about the course and how effective students perceived it to be in attaining its goals. We imported one survey’s content, the general overall course evaluation, from other NJSS CTE/WBL online courses. The third and final survey asked about personal beliefs revolving around climate change. This third survey allowed us to gain insight into how 16–18-year-olds in NJ feel about climate change prior to the formal implementation of new NJ K–12 student learning standards regarding climate change, sustainability, and EJ across subject areas in fall 2022 and how well this new NJSS course targeted this age group. We developed and administered an adapted version of the Student Conservation Association’s (SCA) Climate Change Awareness Survey (2021), originally from a SCA nationwide poll for young adults aged 15–25, at the end of the course [34]. Specifically, we amended the SCA survey to fit the needs of our course by removing any politically charged or potentially controversial questions since funding for this course comes from NJDOE and NJSS, which, as public sector entities and employees, must remain as neutral as possible. The 20 questions used from the SCA survey appear in Table 1. Since technically there were no correct answers in this type of survey trying to gain insight into the perspectives of HS students, we deemed the questions answered as correct, granting credit to participating students for completing the SCA survey. We noted credit (pass/fail) manually in Canvas LMS to participating students who completed this survey.

### 2.4. Study Beta-to-Pilot Testing Periods

The internal Rutgers-beta testing period ran through December 2021 with volunteer testers from NJSS, Rutgers graduate students, and Rutgers undergraduate students. NJSS is covered by an IRB-approved training and evaluation protocol (Rutgers RBHS # 021997W0383).

Names and Rutgers-assigned student email addresses were submitted to Canvas so an account could be created and an invitation to accept the course would be delivered, by Rutgers email, to each volunteer. Each enrolled student needed to accept the course invitation themselves to begin the course. The Rutgers-beta data was collected to see if any edits were needed before pilot testing in NJ HS began.

After this successful preliminary round, NJSS contacted HS throughout NJ, and asked them to volunteer their students to take this free, online, asynchronous course. Three agreed as detailed above: one in central NJ (SCVTHS) and two in southern NJ (CHS, WDHS). Some teachers mentioned they would also offer extra credit to each student who completed the course. A group of Rutgers undergraduate students taking a Byrne Seminar course through the Rutgers Honors College, offered only to first-year college students or freshmen, were also enrolled in the pilot testing in January 2022. Invites were sent out in a similar fashion to those of the Rutgers-beta test group. The HS students had from February to March 2022 through 1 to 4 April 2022, to enroll and complete the course. The Byrne students completed the course materials by 14 February 2022, including evaluation and survey data collection. Teachers and students received reminders to continue accepting the course invite and finish the course throughout the testing period. On 7 April 2022, we exported the course activity and evaluation data into Microsoft Excel and then analyzed the grade information and completion status from the Rutgers Canvas LMS. Descriptive statistics compared Rutgers-beta testers with those from the Byrne seminar undergraduate students and NJ HS students. Of those that did not finish the entire course, there were variations in parts completed and parts not completed. To limit discrepancies, we excluded students who completed ≤2 knowledge checks and did not complete the climate change survey. If they had only finished one of the course surveys, not including the climate change survey, then grades reflected point reductions. Completing the course required taking each of the five (5) modular knowledge checks and completing at least three of them entirely, taking at least one of the two course evaluation surveys, and finishing the SCA-adapted climate change survey.

## 3. Results

During December 2021, two out of two internal testers (NJSS employees completing every aspect of the course), eight out of eleven Rutgers MPH candidates, one out of three other Rutgers graduate students, and one out of five Rutgers undergraduate students completed the course under the group name “Rutgers-Beta” (12/21, 57%). Then, in winter 2022, Rutgers University undergraduate Byrne Seminar students enrolled on 7 February; other enrollment began on 24 February for CHS (S. NJ-1) students and 28 February for WDHS (S. NJ-2) and SCVTHS (C. NJ-1) students. We collected data through 14 February for Byrne Students but until 7 April for the HS groups, though most finished before then, by 1–4 April. Overall, 6 Byrne students were enrolled and 5 finished (83%); 35 CHS (S. NJ-1) students were enrolled and 22 finished (63%); 42 SCVTHS (C. NJ-1) students were enrolled and 31 finished (74%); and 51 WDHS (S. NJ-2) students were enrolled and 29 finished the course (57%). Therefore, overall, 64% (99/155) of students enrolled completed the course.

The pilot testing groups scored, on average, above 90% for the course (Table 2), which entailed completing at least three (3) of the five (5) knowledge checks, at least one of the two course evaluation surveys, and the SCA-adapted climate change awareness survey. The course goal was ≥70% based on NJDOE approval. CHS (S. NJ-1) and Byrne Students scored on average the highest overall at 99.4% ± 0.5% and 99.7% ± 0.4%, respectively. WDHS (S. NJ-2) had the lowest average score, 91.3% ± 14.0%, but still >90% and with low individual student scores >70% when students did not complete two course surveys.

An analysis of the individual knowledge checks provides insight into the success of the students and this course (Table 3). The individual knowledge checks showed a similar trend between schools and groups. Byrne seminar students and Rutgers undergraduate students scored nearly perfect scores on four of five knowledge checks, while CHS’s (S.NJ-1) average score on two of five knowledge checks rounded up to a perfect score. Knowledge check #2 (Climate Change) had the lowest average score between the groups at 4.2 ± 0.2 out of five (5) points. Knowledge checks #3 (Natural Disasters and Extreme Weather Events) and #5 (Environmental Justice Concepts) had the highest at 4.9 ± 0.3 out of five (5) and 4.8 ± 0.3 out of five (5) points, respectively. WDHS (S. NJ-2) contributed the lowest scores observed in the pilot testing, for knowledge check #2 at 3.6 ± 1.0 (Climate Change) and for knowledge check #4 (Sustainability) at 3.6 ± 1.3, but again, these were passing scores of >70% on the knowledge checks.

We also summarized details from knowledge check #3 about specific topics (Natural Disasters and Extreme Weather Events) with relevance to both environmental public health and occupational safety and health in indoor and outdoor locations. This is because of the concerns about heat stress/heat-related illness, and cold stress [30,31,32,33]. Overall, the majority of HS students understood how cold stress is when the temperature of the skin and body starts to drop due to cold weather (96%), how heat stress—potentially leading to exhaustion, illness, stroke, or even death [30,31,32,33]—is when the body undergoes stress from overheating (97%), and how drinking water is one of the best ways to cure a heat-related illness (96%). Moreover, most HS students (88%) also understood how climate change and extreme weather events are related.

We also conducted descriptive and cross-tab analyses of the SCA-adapted “climate change awareness survey” for each of the 20 questions (Table 4) as well as specific groups of related questions pertaining to EJ (Tables S1 and S2 in Supplementary Materials provided online) or environmental pollution or degradation (data were not presented since descriptive results were similar for four of the five major environmental issues, or questions #11–15 of the SCA survey, as summarized below instead of being presented in Table 4). The data provided insights and highlighted what adolescents thought about these environmental pollution-related topics immediately after completing this introductory course.

Most participating HS students believed climate change is real and happening (93.4%), is mostly caused by human or anthropogenic activities (96.1%), and will impact their lives more over the next five years (60.5%) than during the first full school year (2020–2021) of the ongoing COVID-19 pandemic (50.0%). Most participating students are familiar with, i.e., believe in EJ (83.3%); two-in-three (66.7%) students view EJ as part of conserving our environment, but also note that the U.S. could improve the characterization of EJ (55.3%). Over four-in-five participating HS students identified four current environmental issues as major problems, compared to minor problems, with respect to climate change: greenhouse gases and water pollution (81.6% each); toxic materials (86.8%); and deforestation (88.2%). Noise pollution was viewed more as a minor problem (56.6%) than a major problem (43.4%) by participating HS students. Nevertheless, nearly three-in-five participating students (58.3%) also stated their belief that there is still time to prevent the worst effects of climate change, while 13.9% believe it is too late. Participants were nearly split on the question regarding whether this requires major sacrifices (45.8%) compared to minor sacrifices (50.0%) by individuals.

Two-way and three-way cross-tab analyses further confirmed the initial descriptive statistics about the familiarity with and current U.S. characterization of EJ among the participating students. For example, two out of five (40.3%) participants who self-reported familiarity with EJ stated EJ could have an improved characterization and understanding in the U.S., while 36.2% stated the overall characterization and understanding of EJ in the U.S. was generally good regardless of self-reported familiarity with EJ (30.6% if familiar, 5.6% if not familiar). Additionally, twice as many students who believed they had familiarity with EJ thought that conserving the environment was part of EJ than those who did not.

## 4. Discussion

The goal of our online course, “Introduction to HS Students to Climate Change, Sustainability, and Environmental Justice,” delivered statewide in the 2022–2023 school year after the initial testing process summarized in this paper and NJDOE acceptance and approval in May 2022, is to help introduce NJ HS students, especially those in career and technical education, to concepts of climate change and extreme weather (heat and cold), sustainability, and EJ. This course’s content used multiple federal and state resources as credible information to provide a complete, accurate introductory asynchronous course for HS students in support of the educational goals of the U.S. Department of Education and NJDOE, plus other relevant agencies. This course also provided various supplementary resources or materials to HS students on EJ as well as outdoor and indoor microenvironments to increase awareness of related issues such as air quality, water quality, and environmental quality parameters such as temperature and humidity. Advancing, promoting, and expanding available climate change and EJ education is crucial for future generations in the State of NJ, the U.S., and the world. With enhanced education, students will be better prepared for their independent adult lives and future careers, i.e., equipped to enter the workforce to help create a healthier and more sustainable future for modern society. With the new Climate Change Education mandate in the State of NJ by First Lady Tammy Murphy and acceptance of the changing environment due to anthropogenic forces worldwide, implementing and improving the education of environmental science is no longer an optional part of K–12 school curriculums. Educating the younger generations is one of the many crucial steps needed to combat climate change and promote a healthier and more sustainable environment for years to come.

Of the students enrolled from December 2021 to April 2022, 64% finished the entire course. Those that did not finish either finished partially or not at all, which is not an indicator for the success of the course and more so shows their inclination either to take an accessory course or not meet the pilot test phase’s deadline for NJSS. The teachers of students who did complete the course were given an official certificate of completion with 2.0 professional development units (PDUs) to count towards their NJ requirements (100 PDUs every five (5) years). These teachers also noted they offered extra credit to their students for taking the course.

The course offers a short, introductory overview of environmental science, climate change, and sustainability. We graded the course for completion. Because of the variability in the level of completion by the students, looking at the success seen in the individual knowledge checks explains how well the students had learned the subject material. The lowest knowledge checks average score, across groups, was 4.2 out of 5, or 84% for knowledge check #2 (climate change), which is still above NJSS and NJDOE’s goal of >70%. The highest score was 4.9 out of 5, or 98%. These findings are promising for the future success of the course and, consequently, for the ability to teach HS students about the impacts of climate change within environmental science education. This is consistent with other recent research among HS aged students calling for increased climate change education in school science curricula [12,13,14,15], e.g., beyond one lecture in one course or short-course [14,15]. For example, in a study of 188 students ages 16–17 in Austria and Denmark, one lecture in one course only increased assessed knowledge for 1-in-9 students, and overall assessment scores were still below a passing grade (<60%) [14]. In the future, potential NJ HS student gender disparities could also be examined [13,16]. For example, a recent study of HS aged students in 14 public schools in low-income Western Cape areas of South Africa reported 72% knew climate change led to higher temperatures, 60% agreed human activity drove climate change, and 58% believed climate change affects human health; however, males had more knowledge than females [13]. In the future, this NJ HS course could also incorporate activities with interactive visuals—beyond the resources, fact sheets, and infographics currently used—to connect human and ecological health impacts of climate change [17] and even suggest external opportunities available, such as virtual reality simulations, which have helped HS students understand climate change impacts on the environment [18].

A survey in October 2022 by the EdWeek Research Center of about 1000 American adolescents suggested that 3-in-10 HS students in career-technical-vocational education, regardless of their post-secondary education plans, wanted to learn both more about climate change and the jobs that would be available due to it, but only 2-in-9 learned about these from their HS teachers [39]. The data support the importance and value of this study and efforts in NJ.

Further analysis and grading of the course when released to HS across NJ to increase efficiency could see enhancements. After the beta- and pilot test phases, there is no need to have an open-ended question on each of the knowledge checks. Most students answered to the best of their ability, but it was clear, for others, that they did not care to answer and only put down “I don’t know” or “I don’t care” as data entries. This can be challenging to grade with numerous participants. For statewide use of the course, only having multiple choice questions would suffice to show increased immediate impact, i.e., knowledge and application of the material.

### 4.1. Limitations

This project had known limitations. There was a lack of information on whether the NJSS course participants currently had jobs outside of school at the time of the pilot testing from winter to early spring 2022, and the type of work performed. This may have affected their viewpoints due to the type of industry, work, and interactions with co-workers beyond their families and friends at school/on campus. Additionally, NJSS did not investigate the reason for the variability in knowledge check scores among the three groups of pilot test students. NJSS IRB approval for educational training evaluation surveys and module activities/knowledge checks requires anonymous responses that are then deidentified into aggregate analyses. Nevertheless, after the pilot testing period, NJSS decided the questions in knowledge checks should not use open-ended questions but instead only those questions with specific answers (multiple choice and/or Likert-scale responses). This made it less difficult to grade and to evaluate the amount of information they immediately learned from the course. Finally, this study had a small sample size, so the results are not generalizable beyond HS in the State of NJ but can inform future research with youth.

### 4.2. Strengths

This project also had known strengths. This initiative highlights how adolescent/young adult or young worker environmental and occupational health and safety awareness can be increased through free topical online/virtual courses on specific topics, which enhance in-person learning. NJSS spotlighted the influence of heat-related illness on workers at young ages.

## 5. Conclusions

The New Jersey Safe Schools Program’s (NJSS) online course introduces high school (HS) students, especially those in career and technical education, to concepts of climate change and extreme weather (heat and cold), sustainability, and environmental justice (EJ). This course’s evaluation data and HS participant module activities/knowledge check scores suggested initial success during statewide pilot testing in winter-spring 2022, allowing for agency approval and public release to NJ HS spring-summer 2022 for the school year 2022–2023. The course’s module activities/knowledge checks with multiple choice answer options will be available for future evaluation data analyses with potential statistical significance tests with larger sample sizes to compare regions and counties and potentially gender and grade levels. Future research and training program evaluation would examine the course’s broader use in NJ and, pending funding, in other U.S. states in partnership with school districts, universities, and agencies.

## Figures and Tables

**Table 1:** List of questions and answers, “Climate Change Awareness Survey”, adapted by the New Jersey Safe Schools Program (NJSS) from the U.S. Student Conservation Association (SCA) [34].

Questions	Answers				
Do you believe world’s climate is changing?	Yes	No			
Climate change: Is that based more on your personal experiences and observations or more based on what you have read or heard?	Personal experiences and observations	Based on what you have read	Never heard before		
How would you characterize climate change?	Crisis	Problem	Concern	Non-issue	Fiction
Do you believe that human activity is the leading cause of climate change?	Yes	No			
Which of the following actions do you take on a regular basis to lessen your impact on our climate?	Curtail electrical use	Use alternate transportation	Reduce reliance on single-use plastics	Eat vegetarian	Plant trees
When it comes to your own personal actions as they relate to climate change, which of the following comes closest to describing you?	I can and I am making a positive difference	I can make a positive difference, but I am not sure how	There is little I can do, but I wish I could do something	There is little I can do and I am fine with that	Undecided
How confident are you the world will successfully address the causes and consequences of climate change? You are…?	Very Optimistic	Somewhat optimistic	Up in the air	Somewhat pessimistic	Very pessimistic
Which do you believe will impact your life more going forward?	The past year of COVID-19	Next five years of climate change			
How would you characterize your familiarity with environmental justice (EJ)?	Very familiar	Somewhat familiar	Not familiar at all		
How would you characterize EJ in the U.S.?	Very Strong	Generally good	Could use improvement	Extremely inequitable	
What about specific environmental issues: Do you believe greenhouse gases are a …?	Major problem	Minor problem			
Do you believe water pollution is a …?	Major problem	Minor problem			
Do you believe deforestation is a …?	Major problem	Minor problem			
Do you believe noise pollution is a …?	Major problem	Minor problem			
Do you believe the presence of toxic materials is a …?	Major problem	Minor problem			
Rating climate change versus other issues: Would you say addressing climate change is far more important, somewhat more important, about the same in importance, somewhat less important, or far less important than rebuilding the economy?	Far more important	Somewhat important	About the same in importance	Somewhat less important	Far less important
					Undecided
Do you think there is still time to prevent the worst effects of climate change, or too late?	Still time	Too late	Undecided		
Do you think preventing the worst effects of climate change will require major sacrifices from people like you, minor sacrifices from people like you, or no sacrifices at all?	Major sacrifices	Minor sacrifices	No sacrifices		
Is environmental justice a part of conserving our environment or a separate issue?	Conserving our environment	Separate issue unto itself			
On a leisurely or recreational basis, how much time do you spend outdoors per week—would you say <1 h, between 1 and 5 h, between 5 and 10 h, between 10 and 15 h, or >15 h?	Less than 1 h	Between 1 and 5 h	Between 5 and 10 h	Between 10 and 15 h	More than 15 h

**Table 2:** Overall Grades.

Study Sub-Group	Average Grade (%) ± Standard Deviation
Byrne Students	99.7% ± 0.4
CHS (S.NJ-1)	99.4% ± 0.5
Rutgers-Beta	98.5% ± 2.1
SCVTHS (C.NJ-1)	98.9% ± 3.1
West Deptford (S.NJ-2)	91.3% ± 14.0

**Table 3:** Knowledge Check Results.

Study Sub-Group	Knowledge Check 1	Knowledge Check 2	Knowledge Check 3	Knowledge Check 4	Knowledge Check 5
Byrne Students	5.0 ± 0.0	4.3 ± 0.8	5.0 ± 0.0	5.0 ± 0.0	5.0 ± 0.0
CHS (S. NJ-1)	4.7 ± 0.6	4.3 ± 0.7	4.9 ± 0.3	5.0 ± 0.2	5.0 ± 0.2
Rutgers-Beta	4.1 ± 0.8	4.4 ± 0.7	4.8 ± 0.4	4.9 ± 0.3	4.8 ± 0.4
SCVTHS (C. NJ-1)	4.5 ± 0.9	4.5 ± 0.6	4.9 ± 0.2	4.9 ± 0.3	4.8 ± 0.6
WDHS (C. NJ-1)	3.8 ± 1.1	3.6 ± 1.0	4.6 ± 0.8	3.6 ± 1.3	4.4 ± 0.8
*Total Scores*	*4.4 ± 0.5*	*4.2 ± 0.2*	*4.9 ± 0.3*	*4.7 ± 0.5*	*4.8 ± 0.3*

**Table 4:** List of questions and answers to a portion of the “Climate Change Awareness Survey” by the Student Conservation Association, as adapted by NJSS.

	Total (n out of N = 76)	%
* Q #1: Do you believe the world’s climate is changing?		
Yes	71	93.4%
No	4	5.3%
**Q #2: Is [Q1 answer] based more on your…?** *(select all that apply)*		
Personal experiences and observations	42	55.3%
Based on what you have read	59	44.7%
**Q #3: How would you characterize climate change?** *(select all that apply)*		
Crisis	45	59.2%
Problem	41	54.0%
Concern	41	54.0%
Non-issue	9	11.8%
Fiction	7	9.2%
** Q #4: Do you believe that human activity is the leading cause of climate change?		
Yes	73	96.1%
**Q #5: Which actions do you take on a regular basis to lessen your impact on our climate?** *(select all that apply)*		
Curtail electrical use	30	39.5%
Use alternate transportation	30	39.5%
Reduce reliance on single-use plastics	59	77.6%
Eat vegetarian	22	29.0%
Plant trees	21	27.6%
**Q #6: When it comes to your own personal actions as they relate to climate change, which of the following comes closest to describing you?** *(select all that apply)*		
I can and I am making a positive difference	24	31.6%
I can make a positive difference, but I am not sure how	32	42.1%
There is little I can do, but I wish I could do something	24	31.6%
There is little I can do and I am fine with that	11	14.5%
Undecided	14	18.4%
**Q #7: How confident are you that the world will successfully address the *causes and consequences* of climate change—would you say you are…?** *(select all that apply)*		
Very optimistic	14	18.4%
Somewhat optimistic	22	29.0%
Up in the air	32	42.1%
Somewhat pessimistic	27	35.5%
Very pessimistic	10	13.2%
**Q #8: Which do you believe will impact your life *more* going forward?**		
The past year of COVID-19	38	50.0%
The next five years of climate change	46	60.5%
COVID-19 in the past year (2020–2021) ***and*** climate change over the next five years	8	10.5%
**Q #9: How would you characterize your familiarity with environmental justice (EJ)?**		
Familiar	60	83.3%
Not familiar at all	12	16.7%
**Q #10: How would you characterize EJ in the United States?** *(select all that apply)*		
Very strong	12	15.8%
Generally good	30	39.5%
Could use improvement	42	55.3%
Extremely inequitable	8	10.5%
*** Q #16: Rating climate change vs. other issues: Would you say addressing climate change is far more important, somewhat more important, about the same in importance, somewhat less important, or far less important than rebuilding the economy?		
Far more important	13	18.1%
Somewhat important	31	43.1%
About the same in importance	20	27.8%
Somewhat less important	4	5.6%
Far less important	5	6.9%
Undecided	2	2.8%
**Q #17: Do you think there is still time to prevent worst effects of climate change, or it is already too late?**		
Still time	42	58.3%
Too late	10	13.9%
Undecided	20	27.8%
**** Q #18: Do you think that preventing the worst effects of climate change will require major sacrifices from people like you, minor sacrifices from people like you, or no sacrifices at all?		
Major sacrifices	33	45.8%
Minor sacrifices	36	50.0%
No sacrifices	4	5.6%
**Q #19: To you, is EJ a part of conserving our environment or a separate issue?**		
Conserving our environment	48	66.7%
A separate issue unto itself	23	31.9%
Conserving our environment ***and*** as a separate issue.	1	1.3%
***** Q #20: On a leisurely or recreational basis, how much time do you spend outdoors per week? **Would you say <1 h, between 1–5 h, between 5–10 h, between 10–15 h, or >15 h?**		
Less than 1 h	8	11.1%
Between 1 and 5 h	33	45.8%
Between 5 and 10 h	14	19.4%
Between 10 and 15 h	10	13.9%
More than 15 h	8	11.1%

Notes: N = 76. For questions (Q) with answer options adding up to >100% participants could select all that apply. If Q with answer options adds up to <100%, then specific reasons [see footnotes, e.g., missing data (n = 4, N = 72)]. For Q#11–15 on specific environmental issues, data are presented in the text, not in this table. For Q#9, #16–20, N = 72, due to missing data on 4 students. Percentages may add up to 99.9% or 100.1% due to rounding. * “Possibly” was another answer used by SCA that could be selected, and n = 1, 1.3% selected this option. ** “No” and “Both” are other answers that could be selected; n = 1, 1.3% selected “No” and n = 2, 2.6% selected “Both”. *** For this question, three people selected two answer options: far more important + somewhat important (by n = 1), somewhat less important, and far less important (by n = 2). **** For this question, one person selected two answer options (major and minor sacrifices). ***** For this question, one person selected two answer options.

## Data Availability

This study’s data are secured on computers per the IRB-approved stewardship of the New Jersey (NJ) Safe Schools Program. The datasets used and analyzed during the current study are available from the corresponding author upon reasonable request.

## Supplementary Materials

The following supporting information can be downloaded at: https://www.mdpi.com/article/10.3390/ijerph20031922/s1, Figures S1–S5: 2021–2022 modules #1–5 knowledge checks, with questions and answers, respectively. Table S1: Cross-tabulation analyses of 3 questions within a 20-question survey (see Table 1 and Table 4) specific to environmental justice (EJ) in the United States (U.S.) as answered by participating New Jersey high school students in winter 2022. Table S2: Three-way cross-tabulation analyses of questions (Q) within a 20-question survey (see Table 1 and Table 4) specific to environmental justice (EJ) in the United States (U.S.) as answered by New Jersey high school students in winter 2022. Note: One student answered “yes” or “familiar” to Q#9 but “no” to Q#10 and “both” to Q#19.
