# Supplementary material for: Knowledge, Attitudes, and Awareness of New Jersey Public High School Students about Concepts of Climate Change, including Environmental Justice
Source: Int J Environ Res Public Health. 2023 Jan 20;20(3):1922. doi: 10.3390/ijerph20031922 (PMC9915891; doi:10.3390/ijerph20031922)
Supplement: Supplementary file 1 [file ijerph-20-01922-s001.zip › ijerph-2121167-supplementary.pdf]

Supplementary Materials:
Figures S1-S5. 2021-2022 module knowledge checks questions and answers for modules 1-5, respectively.

Figure S1. Knowledge Check 1 (with correct answers)

Knowledge Check 1

Feel free to reference resources given in this first module. If you struggle to answer these questions it may be best to review the module and resources again.

1 Multiple Choice 1 point

Which is not a sphere of the environment?

○ Biosphere
○ Lithosphere
○ Hydrosphere
◉ Metasphere

2 Multiple Choice 1 point

What is a renewable resource?

◉ A resource that can continuously be renewed.
○ A resource that has a finite supply and can be depleted

3 True or False 1 point

Cyclic Resources can be used continuously with no end

◉ True
○ False

4 Multiple Choice 1 point

How many centimeters (cm) are in one inch?

○ 3
○ 254
○ .254
◉ 2.54

5 Essay 1 point

What about environmental science is the most interesting to you?

Figure S2. Knowledge Check 2 (with correct answers)

Knowledge Check 2

Add Instructions...

1 Multiple Choice 1 point

No place on Earth is colder today than it was 100 years ago.

- [x] True
- [ ] False

2 Multiple Choice 1 point

As average global temperature rises,

- [x] Average precipitation increases
- [ ] Average precipitation decreases

3 True or False 1 point

Wasting less food is a way to reduce greenhouse gas emissions.

- [x] True
- [ ] False

4 Multiple Answer 1 point

Which country is the world's largest emitter of carbon dioxide?

- [ ] U.S.
- [x] China
- [ ] India
- [ ] Russia

5 Essay 1 point

What's the difference between climate change and global warming?

0 words

Figure S3. Knowledge Check 3 (with correct answers)

4/11/22, 7:26 PM Knowledge Check 3

Knowledge Check 3

This will not count towards your final grade. Feel free to reference the previous module and resources.

1 Multiple Choice 1 point

What is cold stress?

- [ ] When you are afraid to live somewhere with colder temperatures
- [x] When the temperature of the skin and body start dropping due to cold weather
- [ ] Stress you get when you have a cold

2 Multiple Choice 1 point

What is heat stress?

- [x] When the body undergoes stress from over heating
- [ ] Being afraid you'll sweat through your t-shirt
- [ ] Not wearing enough protective clothing

3 True or False 1 point

Not drinking water is the best way to cure a heat related illness

- [ ] True
- [x] False

4 True or False 1 point

Climate change and extreme weather events are not related.

- [ ] True
- [x] False

5 Essay 1 point

Explain, in your own words, how you think climate change is/is not related to extreme weather events.

https://rutgers.instructure.com/courses/150294/assignments/1710384?display=full_width 1/2

Figure S4. Knowledge Check 4 (with correct answers)

Knowledge Check 4

Feel free to go back to the module or references to answer.

1 Multiple Choice 1 point

What is maximum sustainable yield?

- [ ] How much you need to take to deplete a resource
- [ ] Outdated type of farming
- [x] The optimum level of growth so to not deplete the resource
- [ ] How much food humans can eat

2 True or False 1 point

The NJ Department of Education believes we need to use up all of our natural resources

- [ ] True
- [x] False

3 Multiple Answer 1 point

What is NOT a sustainable practice?

- [ ] Recycling
- [x] Buying
- [ ] Reusing
- [ ] Reducing

4 True or False 1 point

Energy efficiency is one key to reducing climate change risk

- [x] True
- [ ] False

5 Essay 1 point

What does sustainability mean?

0 words

Figure S5. Knowledge Check 5 (with correct answers)

Knowledge Check 5

Answer to the best of your abilities and feel free to reference the previous module and references.

1 Multiple Choice 1 point

What is environmental justice?

- ( ) Giving more resources to the rich
- (•) Understanding the links between environmental health and lower income areas so to prevent any further injustices from happening.
- ( ) Only lawsuits about the environment
- ( ) Following the migration patterns of animals as a result of climate change.

2 True or False 1 point

Redlining pushed lower income communities into more dangerous and unhealthy areas.

- (•) True
- ( ) False

3 Multiple Choice 1 point

Which is one of the 17 principles of Environmental Justice?

- ( ) Preserve the planet
- ( ) Ethical land use
- ( ) Fundamental right to clean air, food, water, etc.
- (•) All of the above

4 True or False 1 point

New Jersey is the first state to deny permits if it could cause an environmental justice issue.

- (•) True
- ( ) False

5 Essay 1 point

What can you do in your own life to prevent future environmental justice issues?

Table S1. Cross-tabulation analyses of three questions within a 20-question survey (see Tables 1 and 5) specific to environmental justice (EJ) in the United States (U.S.) as answered by participating New Jersey high school students in winter 2022.

		Question 10: Student characterized EJ: …in the U.S. was "very strong."	
		Yes	No
Question 9 on student's self-reported familiarity with EJ	Familiar	6 (8.3%)	53 (73.6%)
	Not Familiar	2 (2.8%)	11 (15.3%)
		… in the U.S was "generally good."	
		Yes	No
Question 9 on student's self-reported familiarity with EJ	Familiar	22 (30.6%)	37 (51.4%)
	Not Familiar	4 (5.6%)	9 (12.5%)
		… in the U.S "could use improvement."	
		Yes	No
Question 9 on student's self-reported familiarity with EJ	Familiar	29 (40.3%)	30 (41.7%)
	Not Familiar	9 (12.5%)	4 (5.6%)
		… in the U.S was "extremely inequitable."	
		Yes	No
Question 9 on student's self-reported familiarity with EJ	Familiar	4 (5.6%)	55 (76.4%)
	Not Familiar	0 (0%)	13 (18.1%)

		Question 19 on if student believed EJ is a part of conserving our environment or a separate issue?	
		Yes	No
Question 9 on student's self-reported familiarity with EJ	Familiar	39 (54.2%)	19 (26.4%)
	Not Familiar	4 (5.6%)	9 (12.5%)

Note: One student answered "yes" or "familiar" to question #9 but "no" to question 10 and "both" to question 19.
Note: For questions #9 and #19, N=72, due to missing data on four students.
Note: Percentages may add to 99.9% or 100.1% due to rounding.

Table S2. Three-way cross-tabulation analyses of questions (Q) within a 20-question survey (see Tables 1 and 5) specific to environmental justice (EJ) in the United States (U.S.) as answered by New Jersey high school students in winter 2022.
Note: One student answered "yes" or "familiar" to Q#9 but "no" to Q#10 and "both" to Q#19.

		Question 19 on if student believed EJ is a part of conserving our environment or a separate issue?			
		Yes		No	
		Question 10: Student characterized EJ in the U.S. was "very strong."			
		Yes	No	Yes	No
Question 9 on student's self-reported familiarity with EJ	Familiar	5 (6.9%)	34 (47.0%)	1 (1.4%)	18 (25.0%)
	Not Familiar	0 (0%)	4 (5.6%)	2 (2.8%)	7 (9.7%)

		Question 19 on if student believed EJ is a part of conserving our environment or a separate issue?			
		Yes		Yes	
		Question 10: Student characterized EJ in the U.S. "could use improvement."			
		Yes	No	Yes	No
Question 9 on student's self-reported familiarity with EJ	Familiar	14 (19.4%)	25 (34.7%)	7 (9.7%)	12 (16.7%)
	Not Familiar	1 (1.4%)	3 (4.2%)	3 (4.2%)	6 (8.3%)

		Question 19 on if student believed EJ is a part of conserving our environment or a separate issue?			
		Yes		Yes	
		Question 10: Student characterized EJ in the U.S. was "generally good."			
		Yes	No	Yes	No
Question 9 on student's self-reported familiarity with EJ	Familiar	18 (25.0%)	21 (29.2%)	11 (15.3%)	8 (11.1%)
	Not Familiar	4 (5.6%)	0 (0%)	5 (6.9%)	4 (5.6%)

		Question 19 on if student believed EJ is a part of conserving our environment or a separate issue?			
		Yes		Yes	
		Question 10: Student characterized EJ in the U.S. was "extremely inequitable."			
		Yes	No	Yes	No
Question 9 on student's self-reported familiarity with EJ	Familiar	3 (4.2%)	36 (50%)	1 (1.4%)	18 (25.0%)
	Not Familiar	0 (0%)	4 (5.6%)	0 (0%)	9 (12.5%)
